# *Moringa oleifera* Lam. Leaf Peptide–Calcium Chelates: Structural Characterization, Stability and Calcium Absorption-Promoting Transport Capability

**DOI:** 10.3390/foods15193496

**Published:** 2026-09-29

**Authors:** Ruijing Gu, Xinyu Wang, Zilin Wang, Yu Zhou, Jiahe Dai, Lei Peng, Yang Tian, Liang Tao

**Affiliations:** 1 College of Food Science and Technology, Yunnan Agricultural University, Kunming 650201, China; 13466065297@163.com (R.G.); 15982264033@163.com (X.W.); wangzilin928@163.com (Z.W.); zy190642@163.com (Y.Z.); daijiahe11@163.com (J.D.); daosan100@163.com (L.P.); 2 Engineering Research Center of Development and Utilization of Food and Drug Homologous Resources, Ministry of Education, Yunnan Agricultural University, Kunming 650201, China; 3 Yunnan Key Laboratory of Precision Nutrition and Personalized Food Manufacturing, Yunnan Agricultural University, Kunming 650201, China

**Keywords:** *Moringa oleifera* Lam. leaf peptide-calcium chelate, structural characterisation, stability, dietary factors, calcium absorption promotion

## Abstract

A novel calcium-chelating peptide, KTFQGPPHG, was identified and screened from *Moringa oleifera* Lam. leaf protein hydrolysate, and further used to prepare a highly stable peptide–calcium chelate (KTFQGPPHG-Ca). The structure of KTFQGPPHG-Ca was characterized, while assessing its chelation stability and calcium transport potential. It was found that the calcium binding capacity of KTFQGPPHG reached 62.401 µg/mg. After chelation with calcium, KTFQGPPHG transitioned from an amorphous structure to a stable crystalline form. Carboxyl oxygen atoms and amino nitrogen atoms on KTFQGPPHG served as binding sites for Ca^2+^. The KTFQGPPHG-Ca demonstrates excellent phosphate stability and thermal stability. In addition, the calcium transport amount of KTFQGPPHG at 30 min and 60 min was significantly higher than that of the CaCl_2_ group (*p* < 0.05). Particularly, the calcium transport capacity at 30 min was 1.40-fold that of the CaCl_2_ group. KTFQGPPHG effectively counteracted the suppressive impact of dietary factors on Ca^2+^ absorption, and likely promoted Ca^2+^ translocation via the TRPV6 calcium channel pathway. The present results offer a theoretical basis for KTFQGPPHG-Ca to serve as a promising calcium source to improve intestinal calcium absorption.

## 1. Introduction

Calcium is one of the essential mineral nutrients for sustaining human life, accounting for approximately 1.5–2.2% of total body mass [1]. About 99% of calcium is distributed in bones and teeth in the state of hydroxyapatite, which maintains structural integrity, while the remainder is extensively engaged in key physiological processes such as muscle contraction and nerve transmission [2]. Calcium deficiency can induce disorders including osteoporosis and osteomalacia, which pose a serious threat to human health [3]. However, the human body is incapable of synthesizing calcium de novo, and dietary intake represents the sole pathway to replenish calcium reserves in the skeleton. Conventional calcium preparations, such as calcium citrate and calcium carbonate, have drawbacks including poor solubility, susceptibility to interference from the gastrointestinal environment and dietary anti-nutritional factors [4,5], resulting in inefficient absorption. Thus, developing calcium supplements with excellent stability and high bioavailability is a key approach to prevent and treat calcium deficiency.

*Moringa oleifera* Lam. (*M. oleifera* Lam.) belongs to the order Brassicales, family Moringaceae, genus *Moringa*. It is a deciduous tree or small arbor, native to the southern foothills of the Himalayas in north-western India, and is now widely cultivated in tropical and subtropical regions worldwide. As shown in Figure S1, this plant possesses a spreading umbrella-shaped crown. It bears tripinnate compound leaves with elliptical leaflets. The flowers are white and fragrant, while its pods are slender and pendulous, containing three-winged seeds. The whole plant exhibits edible, medicinal and forage values [6]. As a novel food resource announced by the Chinese Ministry of Health (Document No. 19, 2012, http://www.nhc.gov.cn/), *M. oleifera* Lam. leaves are rich in protein and mineral calcium. Its peptide fractions display diverse biological activities [7,8], rendering them promising raw materials for the development of calcium-supplementing agents [9].

Food-derived peptides can coordinate with calcium ions through active sites such as amino nitrogen and carboxyl oxygen on peptide chains. They can maintain solubility and electrical neutrality during digestion, thereby reducing precipitation and promoting calcium absorption [10]. Our previous research demonstrated that peptide–calcium chelates prepared from *M. oleifera* Lam. leaf protein hydrolysates can effectively promote calcium absorption in rats, increase bone density, and improve bone formation [11]. This might be linked to the fact that peptides derived from *M. oleifera* Lam. leaf protein hydrolysate can enhance the gastrointestinal digestive stability of calcium ions and boost calcium ion uptake and transport. However, the specific types of peptides within the hydrolysate that exhibit high-efficiency calcium chelation, as well as their structural characteristics, stability, and calcium transport capacity, require further investigation. Therefore, this study aimed to identify whether the sequence-known optimal calcium-chelating peptide, screened from *M. oleifera* Lam. leaf peptides, can form a peptide–calcium chelate with favorable structural characteristics, stability, and calcium transport-promoting capacity.

*M. oleifera* Lam. leaves are rich in protein and nutrients, yet direct consumption suffers from poor taste, low acceptability and utilization. This study aims to address the limitation of insufficient high-value utilization of protein resources under the traditional consumption pattern of *M. oleifera* Lam. leaves. We directionally screen calcium-binding peptides with defined sequences and systematically characterize the structural features, chelating interactions, stability, and molecular mechanisms underlying calcium absorption promotion of their peptide–calcium chelates. In parallel, we combined multi-target virtual screening, simulated gastrointestinal digestion, and proteomic analysis to elucidate the gastrointestinal stability of the peptide, its multi-pathway calcium transport mechanisms (TRPV6, Cav1.3, PepT1), and its epithelial regulatory pathways [12]. In the present study, we employed molecular docking technology to screen novel peptides with calcium absorption-promoting potential from *M. oleifera* Lam. leaf peptides and prepared peptide–calcium chelates. We mainly investigated the structural characteristics of peptide–calcium chelates using chromatographic and spectroscopic techniques. The binding stability of the chelates was evaluated in terms of thermal stability and phosphate stability. An in vitro cell model was established to assess calcium transport. This work further enriches the overall research framework for screening peptides with high calcium-binding affinity, preparing peptide–calcium chelates, exploring chelation interactions, and revealing the molecular mechanism of enhanced calcium absorption. This research delivers theoretical basis and experimental evidence for developing plant-sourced peptide–calcium supplements and related health foods, and expands avenues for high-value exploitation of high-quality *M. oleifera* Lam. leaf protein resources.

## 2. Materials and Methods

### 2.1. Materials and Reagents

Fresh *M. oleifera* Lam. leaf was acquired by Honghe Valley Moringa Industry Co., Ltd. (Honghe, China), collected from May to July. The leaves were washed, drained, dried at 65–70 °C, pulverized, and sieved through a 100-mesh sieve to obtain *M. oleifera* Lam. leaf powder. Alkaline protease (≥200 U/mg) was obtained from Solarbio Science & Technology Co., Ltd. (Beijing, China). Fluo-4 AM, a calcium content colorimetric assay kit and Hanks’ balanced salt solution were obtained from Beyotime Biotechnology Co., Ltd. (Shanghai, China). The AKP assay kit was sourced from Nanjing Jiancheng Bioengineering Institute (Nanjing, China). Oxalic acid and phytic acid were procured from Shanghai Tengfei Biotechnology Co., Ltd. (Shanghai, China). The human colon adenocarcinoma Caco-2 cell line used in this study was purchased from Shanghai Zhongqiao Xinzhou Biotechnology Co., Ltd. (Shanghai, China), catalog number: ZQ0056. All other chemicals and reagents applied in this research were analytical-grade.

### 2.2. Preparation of M. oleifera Lam. Leaf Peptides

Proteins from *M. oleifera* Lam. leaves extracted by ammonium sulfate precipitation, modified from Men et al. [11]. To be concise, *M. oleifera* Lam. leaf powder was reconstituted in distilled water (1:35 g/mL), with pH adjusted to 9.0, then stirred in a 50–60 °C water bath for 120 min. Centrifugation at 3500 rpm for 20 min, the supernatant was collected. A 40% saturation level was obtained by adding solid ammonium sulfate to the supernatant, and the mixture was kept standing for a period of 2 h. The precipitate was collected by centrifugation at 3500 rpm for 30 min, washed twice with distilled water, and dialyzed for 6 h in a 3500 Da dialysis bag (water was replaced every 2 h). The sample inside the dialysis bag was collected, freeze-dried to obtain *M. oleifera* Lam. leaf protein, and stored hermetically at 4 °C.

Peptides were prepared following the method of Lin et al. [13]. *M. oleifera* Lam. leaf protein was dissolved in distilled water to prepare a 3% (*w*/*v*) mixture. The solution was heated to 50 °C in a water bath, and the pH was adjusted to 9.0. Alkaline protease (4000 U/g) was supplemented, and enzymatic hydrolysis proceeded for 4 h, heating at 100 °C for 10 min was applied to terminate the reaction. The hydrolysate was centrifuged at 4000 r/min for 30 min with a 1 kDa ultrafiltration tube. The filtrate was lyophilized to obtain the 1 kDa peptide fraction and stored at 4 °C.

### 2.3. LC-MS/MS Identification of M. oleifera Lam. Leaf Peptides

*M. oleifera* Lam. leaf peptides were identified and analyzed using a LC-MS system in positive electrospray ionization (ESI+) mode [14,15]. The detection was performed in positive ion mode, with the precursor ion scanning range set at 300–1800 *m*/*z*. Mobile phase A was 0.1% aqueous formic acid, and mobile phase B was 0.1% formic acid in 84% acetonitrile-water. After equilibrating the column with 95% mobile phase A, the sample was loaded via an autosampler onto a trap column, separated chromatographically, and then analyzed by high-resolution mass spectrometry. By applying a high electric field, electrical charging was induced in the droplets exiting the capillary at the MS inlet. The entire procedure was performed repeatedly until the droplets formed a fine spray, at which point the analyte ions were desorbed and entered the mass analyzer as singly or multiply charged ions.

The parameters for the first-dimensional mass spectrometry (MS^1^) were set as follows: a resolution of 70,000 was adopted at an *m*/*z* of 200, the target level of automatic gain control (AGC) was configured to 16, the upper limit for injection time (Maximum IT) was constrained to 50 ms, and the duration assigned to dynamic exclusion was set at 30.0 s. For the second-stage mass spectrometry (MS^2^), the parameters were configured as: a resolution magnitude of 17,500 at a mass-to-charge ratio of 200 *m*/*z*, an underfill ratio maintained at 0.1%, and normalized collision energy (NCE) of 27 eV. Peptides and their fragment ions were analyzed to obtain their mass-to-charge ratios (*m*/*z*) through the following approach: 20 fragment spectra (MS^2^ scans) were collected after each full scan, with HCD (higher-energy collisional dissociation) as the MS^2^ activation type and and a 2 *m*/*z* width for the ion isolation window. Raw data obtained from mass spectrometry were analyzed via the MaxQuant 1.6.14 search engine and matched to the corresponding protein database retrieved from the UniProt platform (https://www.uniprot.org) [16,17]. Finally, the molecular masses and amino acid sequences of *M. oleifera* Lam. leaf peptides were obtained.

### 2.4. Molecular Docking

Based on previous studies, *M. oleifera* Lam. leaf peptides with a molecular weight (*M*_w_) below 1 kDa demonstrate the most potent calcium-binding ability. Thus, peptide fragments with *M*_w_ < 1 kDa were chosen for molecular docking with the TRPV6 calcium ion channel protein to identify peptides possessing strong binding. The execution of molecular docking was achieved via AutoDockTools-1.5.6. TRPV6 protein 3D structure (PDB ID: 6BO8) was downloaded from RCSB Protein Data Bank (PDB, https://www.rcsb.org/), water molecules and small molecules ligands were excluded in PyMOL_2.6, and hydrogen atoms were added along with charge assignment using AutoDock’s Gasteiger force field [18,19]. The peptide structures were constructed in PyMOL_2.6 according to their amino acid sequences. Blind docking was adopted. The grid box center coordinates were set to x = 100.062, y = 99.87, z = 103.656, with a grid size of 126.0 Å × 126.0 Å × 126.0 Å to cover the overall spatial structure of the TRPV6 protein. The TRPV6 receptor was treated as a rigid body, while the peptide ligand was defined as a flexible molecule. The rotatable torsions and rotatable bonds of the ligand were defined, with exhaustiveness set to 10 and the number of output conformations (num_modes = 9) set to 9. The optimal conformation was selected mainly based on the lowest binding free energy. Docking results were displayed through Discovery Studio 2019 Client and PyMOL_2.6.

### 2.5. Preparation of Peptide–Calcium Chelates

The peptide screened via molecular docking was synthesized by Shanghai Sangon Biotech Co., Ltd. using the Fmoc solid-phase synthesis method. Its purity was verified by HPLC, reaching 95%. Deionized water was used to dissolve the synthetic peptides, and a 1 mg/mL solution was thereby prepared. Subsequently, CaCl_2_ was added with an initial feeding mass ratio of peptide to calcium of 1:1, and the pH of the mixture was adjusted to 7.0. The mixture was then shaken at 50 °C for 60 min. To remove free calcium ions and unreacted peptides, the mixture was added to nine volumes of absolute ethanol. After centrifugation (10,000 rpm, 10 min, 4 °C), the obtained precipitate was recovered and lyophilized, giving peptide–calcium chelates.

### 2.6. Assay for Calcium Binding Capacity

A 1 mL aliquot of the peptide solution (1 mg/mL) was blended with 2 mL phosphate buffer (20 mM, pH 7.8), and 1 mL of CaCl_2_ solution (5 mM) was subsequently added. The mixture was agitated continuously at 37 °C over a 2 h period. Upon cooling to ambient temperature, centrifugation was conducted on the solution at 10,000 rpm for 15 min, and the calcium level within the upper clear phase was determined.

Calcium content was quantified via the o-cresol phthalein complexone (OCPC) colorimetric assay. The mixture in the microplate was thoroughly vortexed, following 5–10 min dark incubation, absorbance was recorded at 570 nm. The calcium-binding capacity was calculated based on the standard curve established in our other study [12].

### 2.7. Structural Characterization

#### 2.7.1. Fourier Transform Infrared Spectroscopy (FT-IR)

First, 2 mg each of the peptides and peptide–calcium chelates were uniformly ground with 200 mg KBr, followed by grinding to homogeneity and pressing into a transparent pellet. The absorption spectra were measured by FT-IR spectrometer (IRTracer 100, Shimadzu Corporation, Kyoto, Japan) over a wavenumber range of 400–4000 cm^−1^, with a resolution of 4 cm^−1^ and 16 cumulative scans. Data analysis was performed using the OMNIC 9.2 software program.

#### 2.7.2. Circular Dichroism Spectroscopy (CD)

Sample solutions were prepared to a concentration of 1 mg/mL. Circular dichroism (CD) spectra were acquired on a Jasco J-1500 spectropolarimeter (JASCO Corporation, Hachioji, Tokyo, Japan) across the wavelength range of 190–260 nm, with a resolution of 0.1 nm and a step size of 1 nm. The raw CD spectra were pre-processed and smoothed with Spectra Analysis 2 software. The relative fractions of secondary structure components were further calculated using the CDNN 2.1 software.

#### 2.7.3. Ultraviolet-Visible Spectroscopy (UV-Vis)

Peptide and peptide–calcium chelate were prepared with deionized water to obtain sample solutions at 1 mg/mL [20]. The UV-Vis absorption spectra of the sample solutions were recorded using a UV-Vis spectrophotometer (SPECORD210plus, Analytik Jena, Jena, Thuringia, Germany) with a 1 cm quartz cuvette, a scan range of 190–400 nm, a scan speed of 50 nm/s, and 1 accumulation. Before sample measurement, blank baseline correction was performed using deionized water.

#### 2.7.4. X-Ray Diffraction (XRD)

For each sample (peptide and peptide–calcium chelate), 30 mg was accurately weighed and ground thoroughly. The measurements were performed using an X-ray diffractometer (Ultima IV, Rigaku Corporation, Akishima, Tokyo, Japan) with current of 40 mA and an accelerating voltage of 40 kV. The sample was scanned from 5° to 90° at a scanning speed of 5°/min.

#### 2.7.5. Fluorescence Spectroscopy (FS)

A Fluoromax-4cl spectrofluorometer (Horiba Scientific, Piscataway, NJ, USA) was employed to investigate the structural conformational variations of the peptides induced by binding to calcium at different concentrations (0, 50, 100, 500, and 1000 mM) [21]. The intrinsic fluorescence of a peptide mainly originates from the aromatic amino acid residues in its sequence: phenylalanine (Phe), tyrosine (Tyr), and tryptophan (Trp). These three amino acid residues have characteristic excitation wavelengths. The excitation peak of phenylalanine in the peptide KTFQGPPHG is approximately 260 nm, and phenylalanine fluorescence can be used to monitor the conformational changes of the peptide induced by calcium binding [22]. After 30 min incubation at 37 °C, fluorescence emission spectra were recorded with an emission spectral range of 300–500 nm and an excitation wavelength set at 260 nm.

#### 2.7.6. ζ-Potential

The samples were dissolved in deionized water (1 mg/mL). The resultant solution was transferred to a U-shaped cell and equilibrated at room temperature for 2 min. ζ-potential measurements were carried out using a ζ-potential analyzer (NanoBrook Omni, Brookhaven Instruments Corporation, Nashua, NH, USA).

#### 2.7.7. ^1^H Nuclear Magnetic Resonance (^1^H NMR)

For each sample (peptide and peptide–calcium chelate), 5 mg was separately dissolved in 0.5 mL of deuterium oxide (D_2_O). The resultant solutions were transferred into 5 mm NMR tubes, and ^1^H NMR spectra were acquired using a nuclear magnetic resonance spectrometer (Bruker-400M, Bruker Corporation, Billerica, MA, USA) at a resonance frequency of 400 MHz.

#### 2.7.8. Scanning Electron Microscopy (SEM)

The surface micromorphologies of the peptide and peptide–calcium chelate were observed using a scanning electron microscope (FEI-Quanta 250, FEI Company, Hillsboro, OR, USA). An appropriate amount of each sample was uniformly spread on conductive adhesive, followed by gold sputtering coating. The observations and micrograph acquisitions were performed at an accelerating voltage of 5 kV, a resolution of 1.06 nm, and a magnification of 500×.

### 2.8. Stability Evaluation

#### 2.8.1. Thermogravimetry–Differential Scanning Calorimetry (TG-DSC)

Thermal stability of the peptides and their calcium chelates were performed by TG-DSC instrument (HITACHI STA200, Hitachi, Ltd., Chiyoda, Tokyo, Japan). Exactly 10 mg of the freeze-dried peptide powder and peptide–calcium chelate powder were separately placed in sealed crucibles. Under a nitrogen atmosphere, the samples were subjected to heating from 30 °C to 900 °C at 10 °C/min, and the corresponding TG-DSC curves were recorded.

#### 2.8.2. Inhibitory Effect on Phosphate Crystallization

The calcium phosphate precipitation inhibition assay was performed with small modifications based on Gao et al. [23]. Briefly, 20 mg peptides were dissolved in 2 mL of 0.8 mol/L CaCl_2_ solution, followed by 5 min incubation in a 37 °C water bath. The mixture was added to 200 mL 0.008 mol/L NaH_2_PO_4_ solution, and the pH was adjusted to 7.4. The pH value was recorded every 5 min intervals until the pH change became relatively stable.

### 2.9. Calcium Transport Assay in Caco-2 Cells

#### 2.9.1. Cytotoxicity Test

Cytotoxicity was evaluated using the MTT assay. Briefly, Caco-2 cells were seeded into 96-well plates at a density of 3 × 10^4^ cells/mL. After incubation for 24 h, the old culture medium was discarded, followed by the addition of 200 μL of the peptides and peptide–calcium chelates at gradient concentrations (0, 50, 100, 200, 400, 800, and 1000 μg/mL) for another 24 h of incubation. Subsequently, after discarding the culture medium, 200 μL MTT solution (0.5 mg/mL) was applied to each well. After 4 h incubation, discard, and 150 μL DMSO supplemented to each well to solubilize formazan crystals. The absorbance at 570 nm was recorded. The cell viability was computed according to Equation (1):

(1) $$\begin{array}{c} \mathrm{cell}\;\mathrm{viability}(\%)=\frac{\left(A_{1}-A_{0}\right)}{\left(A_{2}-A_{0}\right)}\times 100 \end{array},$$

*A*_1_: The absorbance value for the test group; *A*_2_: Control group absorbance value; *A*_0_: Absorbance value of the blank group.

#### 2.9.2. Constructing the Caco-2 Cell Monolayer Model

Caco-2 cells were maintained in RPMI 1640 complete medium containing 1% penicillin-streptomycin and 10% fetal bovine serum (FBS) at 37 °C in 5% CO_2_. Caco-2 cells were inoculated into 12-well transwell inserts (0.4 μm) at 1.0 × 10^5^ cells/well. A volume of 0.5 mL complete medium was added to the apical (AP) side, while 1.5 mL complete medium was added to the basolateral (BL) side, each group was assigned 3 parallel replicate wells. During the initial week after seeding, the complete medium was replaced every other day; thereafter, the medium was refreshed daily until the cells were cultured for approximately 21 consecutive days. The integrity of the cell monolayer model was verified by measuring the transepithelial electrical resistance (TEER) using an EVOM2 epithelial volt-ohmmeter (EVOM2, Beijing Jingong Hongtai Technology Co., Ltd., Beijing, USA). When the TEER reading reached 500 Ω·cm^2^, the established Caco-2 monolayer was judged appropriate for later experiments. The TEER value was calculated using Equation (2):

(2) $$\begin{array}{c} \mathrm{TEER}=\left(R-R_{0}\right)\times S \end{array},$$

*R*: Electrical resistance of the sample well; *R*_0_: Electrical resistance of the blank well; *S*: The 12-well transwell plate possessed an effective membrane area of 1.12 cm^2^.

#### 2.9.3. Calcium Transport in Caco-2 Cells

Upon successful formation of the Caco-2 cell monolayer, the medium in the transwell plates was removed. Following rinsing of the cells with HBSS (2–3 washes), 0.5 mL HBSS was loaded onto the AP side while 1.5 mL HBSS was loaded into the BL side, incubated for 120 min. After removing HBSS, 0.5 mL of different treatment formulations (HBSS, 0.4 mg/mL KTFQGPPHG, 5 mM CaCl_2_, or 0.4 mg/mL KTFQGPPHG + 5 mM CaCl_2_) was individually applied to the AP side. Add 1.5 mL HBSS to the BL side. Sampling of 1 mL HBSS from the BL side was performed at the set incubation time points (30, 60, 120, and 180 min) for calcium content analysis, 1 mL HBSS was replenished at the same time.

#### 2.9.4. Calcium Fluorescence Imaging in Caco-2 Cells

Caco-2 cells were seeded into 30 mm culture dishes at 2 × 10^4^ cells per dish. When the cells reached 80% confluence, they were treated with different test solutions for 12 h: (1) 0.4 mg/mL KTFQGPPHG, (2) 2 mM CaCl_2_, (3) 0.4 mg/mL KTFQGPPHG + 2 mM CaCl_2_, and (4) 0.4 mg/mL KTFQGPPHG + 4 mM CaCl_2_. Subsequently, 200 μL of Fluo-4 AM working solution (4 μM) was added to each dish. Pluronic F-127 was simultaneously added at a final concentration of 0.04–0.05% to enhance the cellular uptake of Fluo-4. The mixture was gently shaken to achieve full cell coverage, and the dishes were maintained in a constant-temperature incubator for 30 min to load the fluorescent probe. Subsequently, cells were rinsed thrice with HBSS buffer, then incubated for 10 min to guarantee the full intracellular transformation of Fluo-4 AM into Fluo-4. Images of cellular fluorescence were captured using an inverted fluorescent microscope (DMI 3000B, Beijing RuiKe ZhongYi Technology Co., Ltd., Beijing, China).

#### 2.9.5. Calcium Uptake Under Dietary Inhibitory Factors

A calcium intake assay was carried out under conditions containing dietary calcium absorption inhibitors, in accordance with the documentedas by Hou et al. [24]. Caco-2 cell monolayers grown on transwell plates were washed 2–3 times with HBSS and pre-incubated for 30 min using HBSS. Then, 0.5 mL of the following solutions loaded onto the AP compartment: (1) 0.4 mg/mL KTFQGPPHG + 5 mM CaCl_2_ + 5 mM dietary factor (phytic acid or oxalic acid), or (2) 5 mM CaCl_2_ + 5 mM dietary factor (phytic acid or oxalic acid). Then, 1.5 mL HBSS was placed into the BL compartment. The calcium level in the BL side was quantified after 2 h incubation.

#### 2.9.6. Investigation of the Calcium Absorption Pathway of KTFQGPPHG-Ca

Transwell plates were seeded with Caco-2 cells at 1 × 10^5^ cells/well density. The cells were washed with HBSS, and re-incubated with HBSS for 30 min. The cells were then pre-incubated with distinct concentrations (0, 6.25, 12.5, 25, and 50 μM) of the 2-APB for 1 h, after which the medium was removed. Next, 0.4 mg/mL KTFQGPPHG + 5 mM CaCl_2_ + diverse concentrations of 2-APB solution were dispensed to the AP side. Following a 2 h incubation period, the calcium content in the BL side solution was assayed.

### 2.10. Statistical Analysis

All experiments were performed in triplicate, with data presented as the mean ± SD. Data analysis was performed using SPSS 23.0 (SPSS 23.0, Inc., Chicago, IL, USA). In this study, one-way ANOVA was used for single-factor experimental data, followed by Duncan’s multiple comparison test. For the Caco-2 monolayer cell calcium transport experiment, two-way ANOVA was employed, with sample treatment group and incubation time as fixed factors. Different uppercase or lowercase letters indicate significant differences among groups (*p* < 0.05), and *p* < 0.05 was considered statistically significant. Three biological replicates were set for the experiments. GraphPad Prism 8 (16.0) and Origin 2021 Pro were used for graph processing.

## 3. Results and Discussion

### 3.1. Screening for the Optimal Calcium-Binding Peptide

Prior research has shown that *M. oleifera* leaf peptides with a molecular mass below 1 kDa possess stronger affinity for calcium ions [25,26]. In this study, a total of 70 peptides with *M*_w_ < 1 kDa were identified and subjected to molecular docking with the calcium ion channel protein TRPV6. TRPV6 is a key protein mediating transcellular calcium transport in the small intestine. Simple peptide–calcium chelation can only maintain the solubility and stability of calcium ions and cannot actively promote intestinal transmembrane absorption. Therefore, in this study, molecular docking-based preliminary screening was performed using TRPV6 as the target, aiming to screen candidate peptides with dual functions of calcium loading and calcium transport promotion from a complex peptide library. As shown in Table 1, molecular docking against TRPV6 revealed that peptide KTFQGPPHG (*M*_W_ = 967.4876 Da) possessed the lowest binding energy (−9.3 kcal/mol). Peptide GWLGHPIF (*M*_W_ = 925.481 Da) yielded a binding energy of −9.2 kcal/mol. Owing to their close docking scores, KTFQGPPHG and GWLGHPIF were chosen for further investigations.

**Figure 1:**
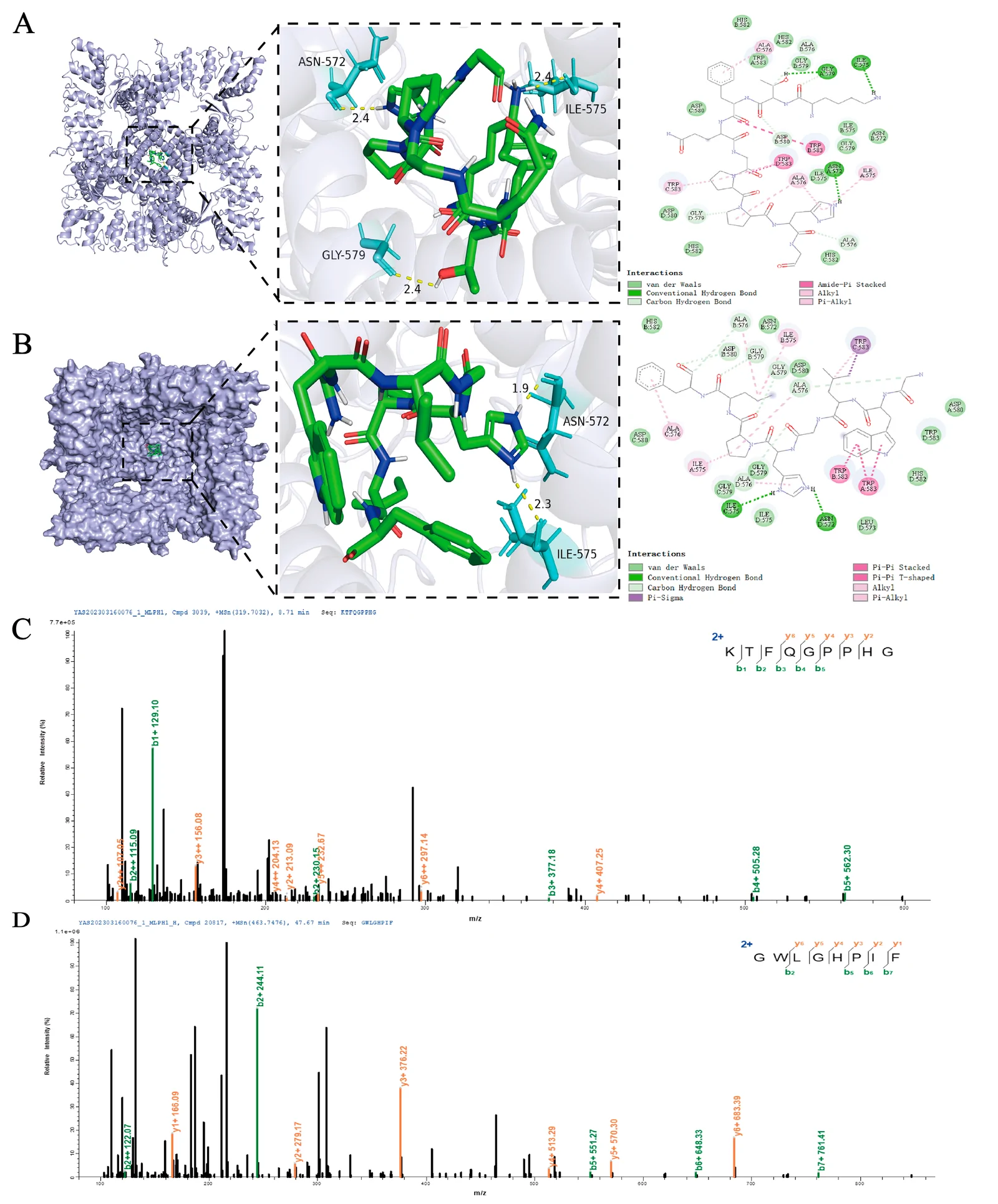
Visualization of peptide-TRPV6 molecular docking and secondary mass spectrometry diagram of peptide segments. (**A**) Three-dimensional and two-dimensional visualization of KTFQGPPHG docked with the TRPV6 molecule; (**B**) three-dimensional and two-dimensional visualization of GWLGHPI docked with the TRPV6 molecule; (**C**) secondary mass spectrometry spectrum of KTFQGPPHG (Reproduced from Wang et al. [12], with permission from American Chemical Society, 2026); (**D**) secondary mass spectrometry spectrum of GWLGHPIF.

As shown in Figure 1A,B, the 3D visualization revealed that in KTFQGPPHG, Lys formed hydrogen bonds with Asn^572^, Thr with Ile^575^, and His with Gly^579^ of TRPV6, with bond lengths of approximately 2.4 Å. In GWLGHPIF, His formed hydrogen bonds with Asn^572^ and Ile^575^ of TRPV6, with bond lengths of 1.9 Å and 2.3 Å, respectively. The number and length of hydrogen bonds are known to predict their strength [27]. Both KTFQGPPHG and GWLGHPIF formed relatively short hydrogen bonds with TRPV6, indicating strong hydrogen bonding interactions. Kim et al. [28] demonstrated that hydrophobic interactions enhance docking stability. According to the 2D visualization, the hydrophobic interactions between KTFQGPPHG and TRPV6 involve Ile^575^, Ala^576^, and Trp^586^. The hydrophobic interactions between KTFQGPPHG and TRPV6 involve Ile^575^ and Ala^576^. In summary, these findings indicate that KTFQGPPHG and GWLGHPIF bind stably to the TRPV6 calcium channel protein primarily through hydrogen bonding and hydrophobic interactions.

The MS/MS spectra of KTFQGPPHG and GWLGHPIF are displayed in Figure 1C,D. HPLC analysis revealed the purity of the synthesized peptides to be in excess of 95% (Figure S2). The calcium-binding capability of KTFQGPPHG and GWLGHPIF were calculated to be 62.40 μg/mg and 58.19 μg/mg, respectively. The calcium-binding potential of KTFQGPPHG was obviously superior to GWLGHPIF (*p* < 0.05). Furthermore, KTFQGPPHG demonstrated the highest binding affinity toward the TRPV6 calcium channel. Consequently, KTFQGPPHG was designated as the peptide for follow-up experiments.

### 3.2. Structural Characterization

#### 3.2.1. FT-IR

**Figure 2:**
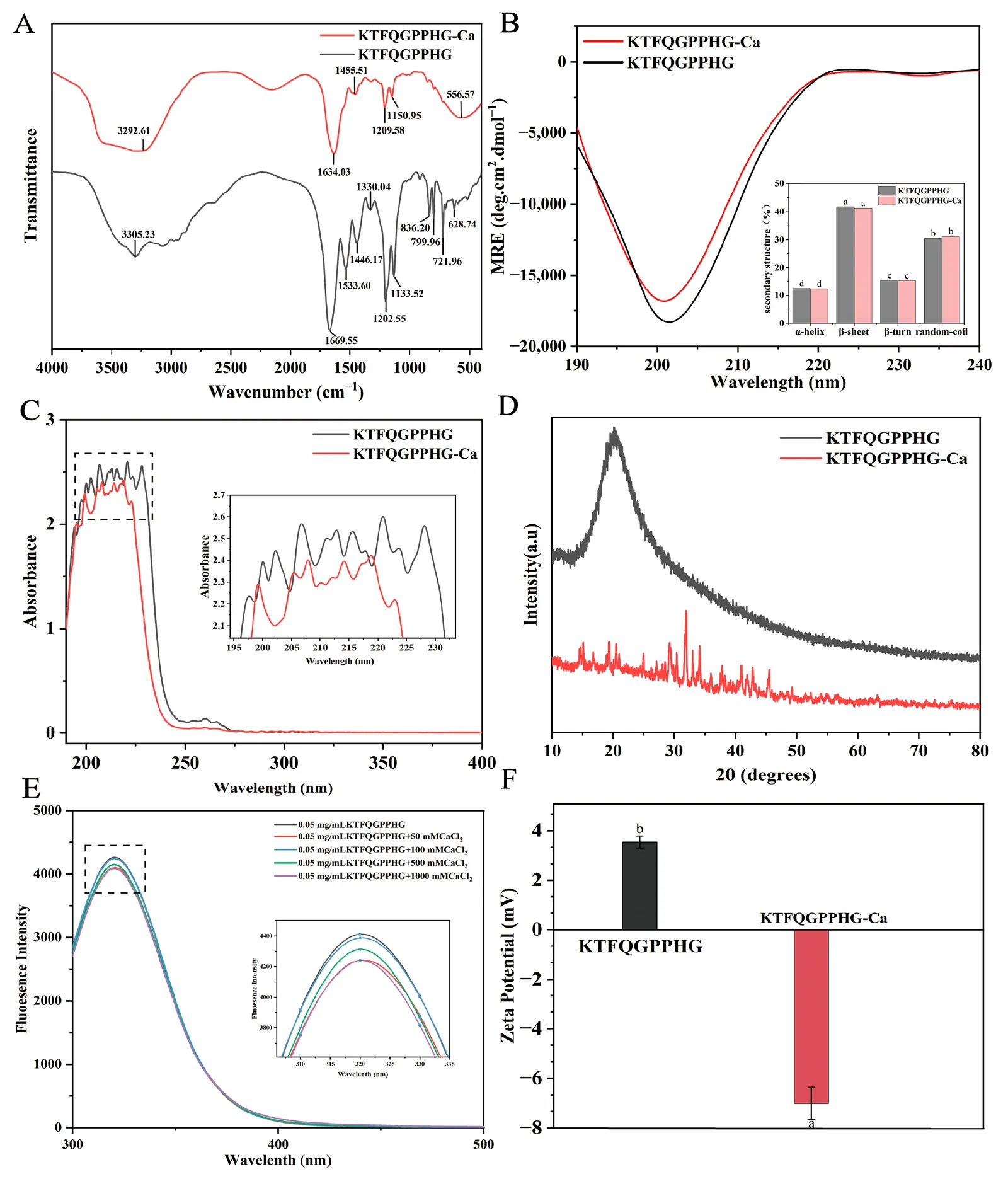
Structural characterization of KTFQGPPHG and KTFQGPPHG-Ca. (**A**) FT-IR; (**B**) CD; (**C**) UV-vis; (**D**) XRD; (**E**) FS; (**F**) ζ-potential. Different superscript letters indicate significant differences among groups (*p* < 0.05).

The FT-IR spectra of KTFQGPPHG and KTFQGPPHG-Ca are shown in Figure 2A. In KTFQGPPHG, the high-frequency absorption at 3305.23 cm^−1^ was attributed to N–H stretching vibrations. Subsequent to calcium chelation, this absorption peak shifted to 3292.61 cm^−1^, suggesting that the electron cloud density of N–H was modified as a result of either an inductive or a dipole field effect [29]. It is speculated that –NH_2_ groups chelated with Ca^2+^ to form ammonium salts. In KTFQGPPHG, the absorption peak observed at 1669.55 cm^−1^ was assigned to the amide I band due to C=O stretching vibrations, while the peak at 1533 cm^−1^ was attributed to the amide II band arising from C–N stretching vibrations [30]. In KTFQGPPHG-Ca, the amide I band exhibited a red shift to 1634.03 cm^−1^, indicating that C=O groups were involved in the peptide–calcium chelation reaction, whereas no significant change was observed for the amide II band. Furthermore, a new characteristic peak emerged at 1455.51 cm^−1^ in the spectrum of KTFQGPPHG-Ca, which is a signature of the symmetric stretching vibration of the carboxyl group [31]. This indicates the coordination of Ca^2+^ with –COOH, leading to the formation of –COOCa. This finding is consistent with the FT-IR spectral results reported by Lin et al. [13] for walnut-derived peptide–calcium chelates. Multiple changes in absorption peaks were observed for KTFQGPPHG-Ca in the low-frequency region of 500 cm^−1^–1000 cm^−1^, which may be related to the diminution in electron cloud density, bond strength, and stretching vibration frequency of the C–O bond caused by peptide–calcium chelation, and the occurrence of chelation may lead to the disappearance of peaks in the low-frequency region [32]. The absorption peak at 556.57 cm^−1^ in KTFQGPPHG-Ca is imputable to the N–Ca stretching vibration, further confirming the involvement of amino groups in the process of chelation. This observation was also reported by Sun et al. [33] in their FT-IR study on deer-tendon peptide–calcium chelates [33]. Differences in spectral peak intensity and wavenumber demonstrated that a chelation reaction occurred between KTFQGPPHG and Ca^2+^, forming a new compound distinct from the original peptide, and that the amino nitrogen atoms and carboxyl oxygen atoms in the peptide are the main binding sites for the chelation of KTFQGPPHG with Ca^2+^.

#### 3.2.2. CD

The secondary structure of proteins can be analyzed by CD spectroscopy [34]. As shown in Figure 2B, KTFQGPPHG exhibited a negative band at 201 nm, which blue-shifted to 200 nm and increased in intensity after the formation of KTFQGPPHG-Ca. KTFQGPPHG was composed of β-turn, β-sheet, random coil structures and α-helix, with random coil and β-sheets being the dominant structure, accounting for 30.47% and 41.72%, respectively. After binding with Ca^2+^, the content of α-helix declined to 12.35%, the β-turn content decreased to 15.28%, the β-sheet content decreased to 41.23%, and the random coil content increased to 31.12%. Peptide–calcium binding increases hydrophobic groups exposure and breaks hydrogen bonds, diminishing α-helices, leading to a more extended conformation of the peptide chain [35]. Covalent bond formation between Ca^2+^ and the KTFQGPPHG peptide triggered structural conformational alterations in the peptide, promoting the conversion of α-helix, random coil, and β-turn into a more ordered, stable conformation. This result is similar to the secondary structure changes of peptide–calcium chelates derived from Pacific sturgeon bone reported by Zhang et al. [36]. CD results indicate that the binding of KTFQGPPHG with Ca^2+^ generates new ionic and coordinate bonds, thereby altering the secondary structure of KTFQGPPHG. This confirms that the interaction with Ca^2+^ yields a new compound with a distinct conformation compared to the original KTFQGPPHG peptide.

#### 3.2.3. UV-Vis

Alterations in the peak position and intensity of UV-Vis absorption spectra can serve to analyze material changes and detect the appearance of new substances [37]. As shown in Figure 2C, the maximum absorption peaks of both KTFQGPPHG and KTFQGPPHG-Ca were observed at approximately 210 nm, which is mainly attributed to the n → π* transition of the carbonyl C=O group in the peptide bond [38]. After the addition of Ca^2+^, the maximum UV absorption peak of KTFQGPPHG shifted to a shorter wavelength, from 221 nm to 208 nm. This may be ascribable to the absorption of certain wavelengths in the UV region by the metal ion and peptide ligands, leading to electronic transitions. Another possibility is that the bonding of Ca^2+^ with the carboxyl oxygen atoms and amino nitrogen atoms in the peptide induces internal electronic transitions of the ligands [39]. After chelation, KTFQGPPHG-Ca exhibited a slight hypochromic effect, with the maximum absorption intensity decreasing from 2.66 to 2.46. This could be attributed to the chelation altering the polarization and polarity of chromophores (–CO, –COOH) and auxochromes (–OH, –NH_2_) [40], resulting in alterations in absorption peak intensity. The differences in the UV characteristic peaks between KTFQGPPHG and KTFQGPPHG-Ca indicate that specific interactions occurred between KTFQGPPHG and Ca^2+^, altering the peptide molecular structure and ultimately forming a new compound, KTFQGPPHG-Ca.

#### 3.2.4. XRD

XRD can reflect the formation of complexes between organic ligands and metal ions. As a method commonly used to analyze changes in molecular crystallinity, it can provide information such as the crystalline state of samples from the diffraction patterns [41,42]. As shown in Figure 2D, significant differences were observed in the XRD peaks between KTFQGPPHG and KTFQGPPHG-Ca. The introduction of Ca^2+^ affected the shape of the diffraction peaks. At a 2θ angle of 20.12°, KTFQGPPHG presented a wide diffraction peak, signifying low crystallinity and an irregular amorphous structure [13]. After binding with Ca^2+^, this broad peak disappeared and was replaced by several sharp, discrete diffraction peaks. This suggests the possible formation of a new, ordered crystalline structure. The changes in diffraction peaks indicate that Ca^2+^ crosslinking induced peptide assembly, transforming KTFQGPPHG from an amorphous state to a crystalline state with further increased crystallinity [13]. Similar changes in crystalline structure were also observed by Hu et al. [43] in their study on the introduction of calcium into sheep bone peptides. The characteristic changes in the shape, intensity, and position of the diffraction peaks in the XRD patterns confirm that a complexation reaction occurred successfully between KTFQGPPHG and Ca^2+^, forming a new compound, KTFQGPPHG-Ca, whose crystal structure differs from that of the original peptide.

#### 3.2.5. FS

Fluorescence spectroscopy can characterize the interaction between organic ligands and metal ions through changes in absorption peaks and fluorescence intensity [44], and aromatic amino acids in peptides can yield endogenous fluorescence at appropriate excitation wavelengths, providing an effective basis for analyzing peptide structural changes [45]. As shown in Figure 2E, with increasing Ca^2+^ concentration in the system, the fluorescence intensity of KTFQGPPHG at 320 nm gradually decreased from 4411 to 4239. In particular, when 50 mM CaCl_2_ was added to the KTFQGPPHG solution, the endogenous fluorescence decreased significantly. Chelation of Ca^2+^ with active groups on the peptide chain, especially amino and carboxyl groups, can induce the aggregation of amino acid residues and the folding of the peptide’s spatial structure, resulting in changes in fluorescence intensity [46]. As the Ca^2+^ concentration increases, Ca^2+^ can bind to aromatic amino acids or interact with chromophores in the peptide to alter excited-state energy, producing a fluorescence quenching effect and thus decreasing fluorescence intensity [47]. Furthermore, with a further increment in Ca^2+^ concentration, the degree of fluorescence quenching gradually slowed, which may be related to the saturation of potential Ca^2+^ binding sites on the peptide chain. A decline in fluorescence intensity constitutes a representative spectral signature of structural modifications when peptides undergo chelation with Ca^2+^ [48]. Therefore, the fluorescence spectroscopy results indicate that KTFQGPPHG chelated with Ca^2+^ to form a new substance.

#### 3.2.6. ζ-Potential

The surface charge status of protein particles caused by amino acid ionization can be characterized via ζ-potential analysis [49]. The ζ-potential is related to the stability of colloidal systems; a higher absolute ζ-potential value indicates greater electrostatic repulsion between particles and better stability [50]. As shown in Figure 2F, the ζ-potential value of KTFQGPPHG-Ca (−7.02 mV) was significantly lower than that of KTFQGPPHG (3.54 mV; *p* < 0.05). It is speculated that this phenomenon arises from the interaction between Ca^2+^ and KTFQGPPHG. Negatively charged carboxyl groups in KTFQGPPHG bind to positively charged Ca^2+^ ions, leading to the neutralization of negative charges on the KTFQGPPHG surface and a consequent change in potential [51]. Previous studies have also reported that Ca^2+^ can shield negative charges on polypeptide chains and act as salt bridges to induce protein aggregation [52]. After the chelation reaction, the ζ-potential changed from positive to negative, indicating that after Ca^2+^ was chelated by the positively charged functional sites in KTFQGPPHG, the resulting KTFQGPPHG-Ca exhibited a net negative charge. The enhanced electrostatic repulsion contributed to improved stability, while particle aggregation was observed during the formation of the peptide–calcium chelate. This is consistent with the results of Huan et al. [10] on the zeta potential of novel peptide–calcium chelates synthesized by enzymatic methods.

#### 3.2.7. ^1^H NMR

**Figure 3:**
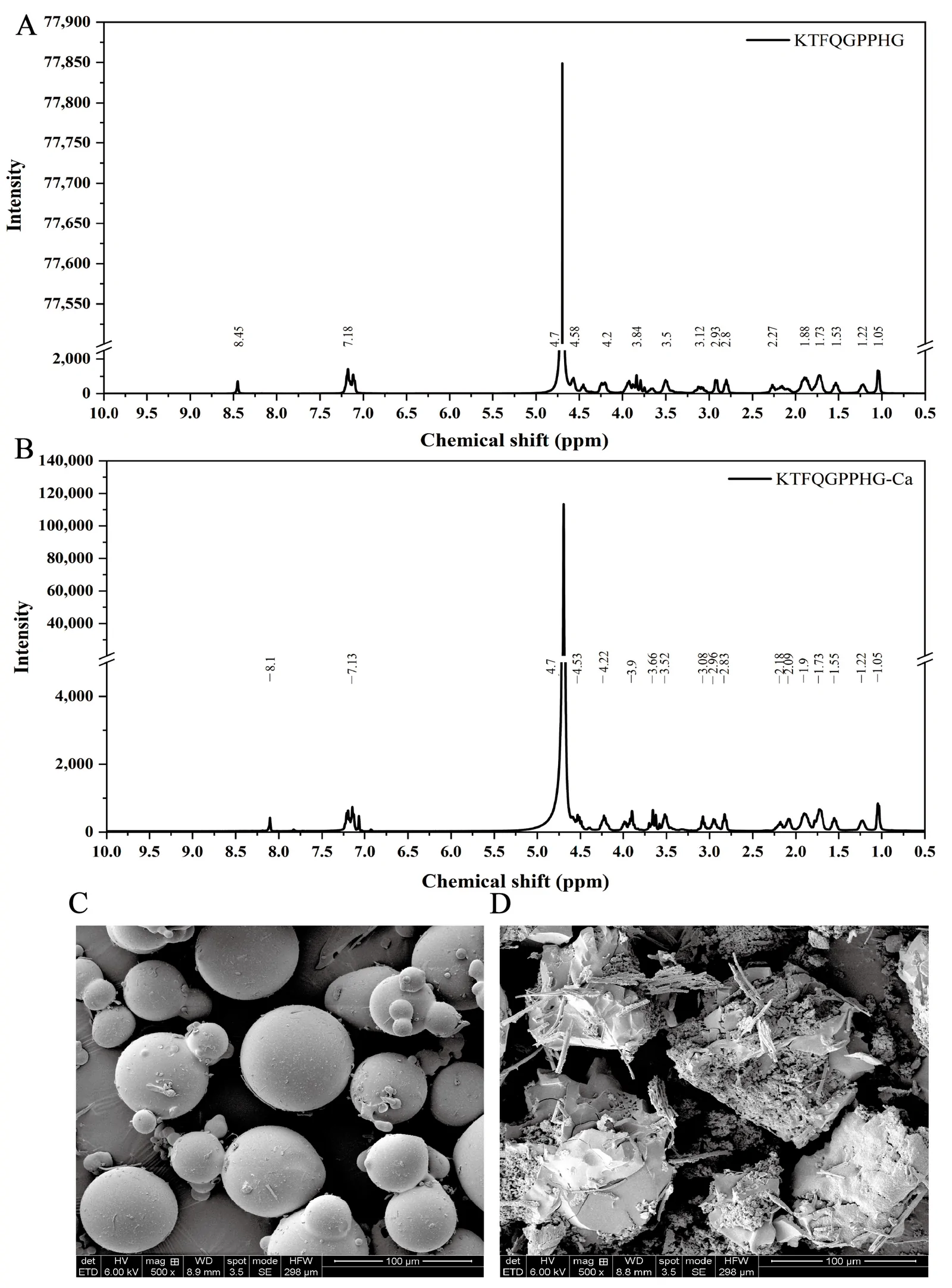
Structural characterization. (**A**) ^1^H NMR spectrum of KTFQGPPHG; (**B**) ^1^H NMR spectrum of KTFQGPPHG-Ca; (**C**) SEM image of KTFQGPPHG; (**D**) SEM image of KTFQGPPHG-Ca.

^1^H NMR spectroscopy can reveal the interaction mechanism between polypeptides and Ca^2+^ by reflecting changes in the electron cloud distribution around hydrogen nuclei, and it is an important technique for analyzing alterations in the chemical environment of hydrogen atoms and the structural characteristics of compounds during chelation reactions [53]. As shown in Figure 3A,B, significant differences were observed between the ^1^H NMR spectra of KTFQGPPHG and KTFQGPPHG-Ca. The peak at 4.7 ppm corresponds to the solvent signal of D_2_O, which remained unchanged during the binding process. Compared with KTFQGPPHG, the characteristic peak intensities of KTFQGPPHG-Ca generally decreased, and multiple peaks exhibited chemical shifts to varying degrees, indicating that the introduction of Ca^2+^ altered the microenvironment of hydrogen nuclei within the polypeptide chain. In the ^1^H NMR spectra, signals at δ ≤ 1.5 ppm are primarily owing to hydrogens of –CH_3_ groups; those in the δ 1.5–3.0 ppm range to hydrogens of –CH_2_ groups; and signals between δ 4.0 and 5.0 ppm to hydrogens bound to strong electron-withdrawing groups (e.g., oxygen) [54]. The changes in peak intensity at 1.5–3.5 ppm are speculated to be related to the spin-coupling splitting of β-H atoms in Lys, Gly, and Phe. After chelation, the characteristic peak near δ = 4.58 ppm shifted upfield and decreased in intensity. The peaks in the range of 3.5–4.7 ppm became broadened, decreased in intensity, and were accompanied by a downfield shift, which may correspond to the spin-coupling splitting of α–H in Phe, Gly, and Lys [38]. In the low-field region, near δ = 7.18 ppm and δ = 8.45 ppm, the peak positions shifted toward the high-field region after chelation. This may be ascribed to the generation of coordinate bonds between the N atoms of –NH_2_ groups and the O atoms of –COOH groups in the peptide with Ca^2+^, which causes the lone-pair electrons to shift toward Ca^2+^, resulting in a relative increase in the electron cloud density of adjacent hydrogen nuclei and thus inducing an upfield chemical shift [55]. Taken together, the bonding of Ca^2+^ to the peptide chain affects the electron cloud environment of the hydrogen protons, leading to changes in the positions of the split peaks resulting from spin–spin coupling of hydrogen protons at different chemical shifts after the chelation reaction between the peptide and Ca^2+^ [56]. The changes in intensity and position of the absorption peaks indicate that the amino and carboxyl groups in the peptide provided coordination sites for Ca^2+^. Ca^2+^ participates in the chelation reaction by substituting hydrogen atoms at different positions within the peptide, and Phe, Gly, and Lys are identified as potential calcium-binding sites.

#### 3.2.8. SEM

SEM is widely used to observe the microstructure of samples. As shown in Figure 3C,D, KTFQGPPHG appeared as spherical particles with a relatively uniform morphology and a loosely distributed structure. After the introduction of Ca^2+^, KTFQGPPHG-Ca exhibited a block-like morphology with a rough surface and numerous attached particle aggregates. This morphological difference may originate from changes in the internal structure of KTFQGPPHG [57]. The XRD analysis results also confirmed this: the coordination bonds between KTFQGPPHG and Ca^2+^-induced folding of the internal structure to generate small granular substances. These particles attracted one another and aggregated, thereby producing a surface-attached granular appearance and leading to changes in the surface morphology of KTFQGPPHG. The small particulate matter may be calcium salt crystals adsorbed on the peptide surface. SEM analysis demonstrated that chelation with Ca^2+^ altered the morphology of KTFQGPPHG, resulting in the formation of the structurally distinct KTFQGPPHG-Ca complex.

### 3.3. Stability Analysis

#### 3.3.1. TG-DSC

**Figure 4:**
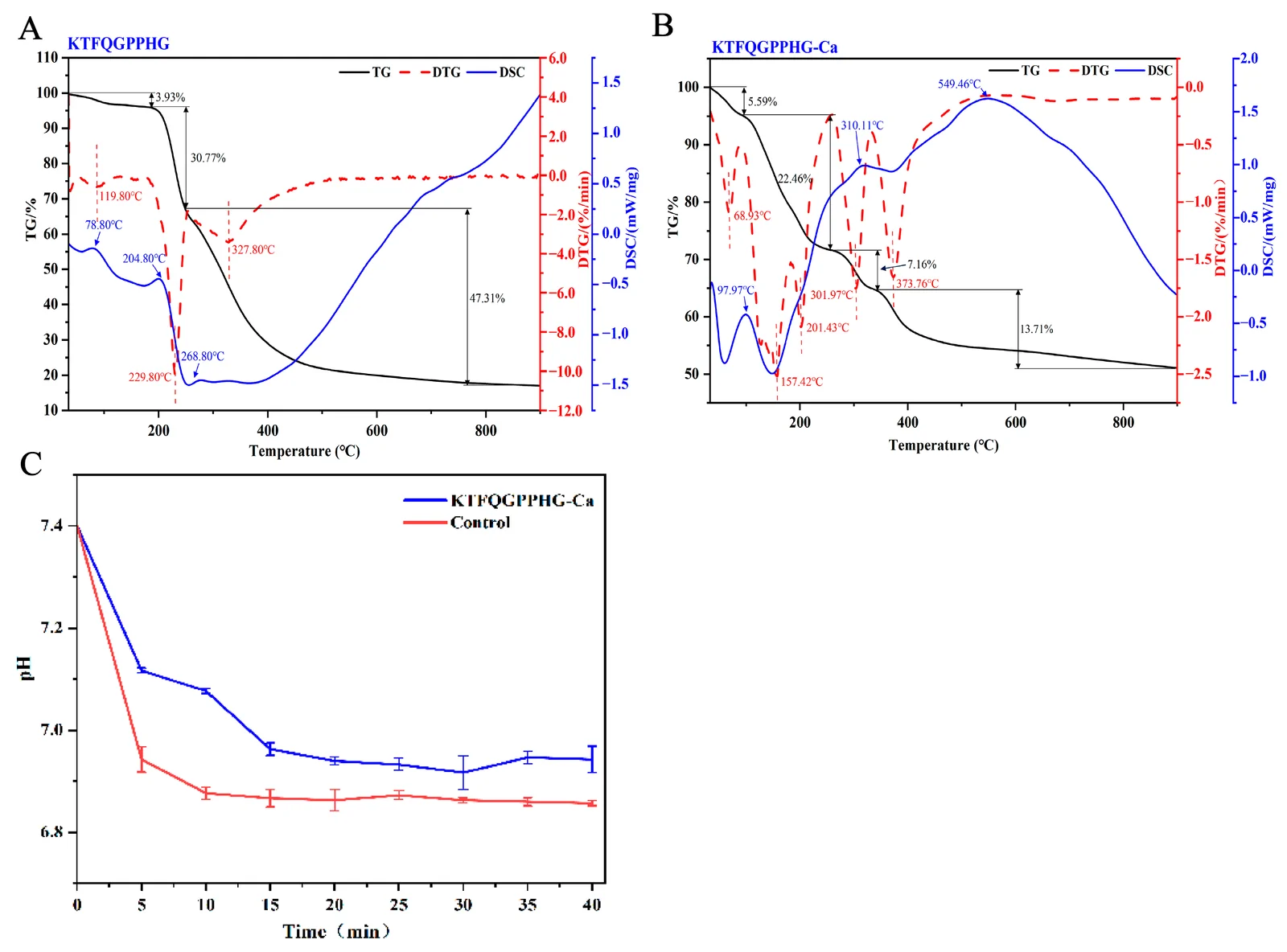
Stability analysis of KTFQGPPHG-Ca. (**A**) TG-DSC image of KTFQGPPHG; (**B**) TG-DSC image of KTFQGPPHG-Ca; (**C**) Phosphate stability.

As shown in Figure 4A, the TG curve of KTFQGPPHG exhibited three weight loss stages: 30–167.80 °C with a mass loss of 3.93%, 167.80–256.80 °C with 30.77% loss, and 256.80–900 °C with 48.31% loss. The total mass loss of KTFQGPPHG was 83.01%. The weight loss at 30–167.80 °C mainly from freezable water (bound and free water) on the peptide chain surface. The weight loss at 167.80–900 °C mainly resulted from peptide bond cleavage, manifested as the amino acid deamination, decarboxylation and polypeptide depolymerization [58]. As depicted in Figure 4B, KTFQGPPHG-Ca displayed four weight loss stages: 30.00–103.77 °C (5.59%), 103.77–245.20 °C (22.46%), 245.20–337.63 °C (7.16%), and 337.63–900 °C (13.71%), with a total weight loss of 48.92%. The weight loss of KTFQGPPHG-Ca between 30 and 103.77 °C is attributed to the same reason as KTFQGPPHG, with the temperature difference possibly indicating variations in water content between the two samples. The organic components of KTFQGPPHG-Ca began to decompose gradually at 103.77 °C. The total mass loss and the rate of weight loss were significantly higher for KTFQGPPHG than for KTFQGPPHG-Ca. Under identical heating conditions, KTFQGPPHG-Ca showed less mass loss and a slower degradation rate, indicating superior thermal stability. The DSC curve of KTFQGPPHG exhibits three absorption peaks at 78.80 °C, 204.80 °C, and 268.80 °C, while the DSC curve of KTFQGPPHG-Ca displays three endothermic peaks at 97.97 °C, 310.46 °C, and 549.46 °C. Following chelation with Ca^2+^, the corresponding endothermic peaks of KTFQGPPHG shifted to the right. This indicates increased bond energy within the chelate structure, conferring greater stability and requiring higher energy to disrupt the bonds [59]. Consequently, KTFQGPPHG-Ca exhibits superior thermal stability.

#### 3.3.2. Phosphate Stability

Phosphate exhibits strong metal-chelating ability and forms poorly soluble calcium phosphate complexes, whose precipitation reduces the bioavailability of intestinal calcium [60]. During calcium phosphate crystallization, the deprotonation of H_2_PO_4_^−^ and HPO_4_^2−^ releases large amounts of H^+^, and the extent of pH decrease can effectively reflect the amount of calcium phosphate crystals formed [61]. The effect of phosphate solution on KTFQGPPHG-Ca is shown in Figure 4C. The pH of KTFQGPPHG-Ca decreased rapidly within the first 15 min and then stabilized at pH 7.0. In contrast, the pH of the control CaCl_2_ solution decreased more significantly within the first 10 min and eventually stabilized at pH 6.8. Compared with CaCl_2_, the pH decrease rate in the KTFQGPPHG system was slower, indicating that calcium phosphate crystallization was inhibited. This may be attributed to KTFQGPPHG providing chelation sites that effectively bind Ca^2+^, thereby preventing the production of insoluble calcium phosphate [62]. The results of the calcium phosphate crystallization inhibition assay indicate that KTFQGPPHG can reduce the production of calcium phosphate precipitates, which could contribute to the enhancement of Ca^2+^ bioavailability in the intestinal tract.

### 3.4. Calcium Uptake and Transport Abilityy in Caco-2 Cells

#### 3.4.1. Establishment of the Caco-2 Monolayer Model and Calcium Transport

**Figure 5:**
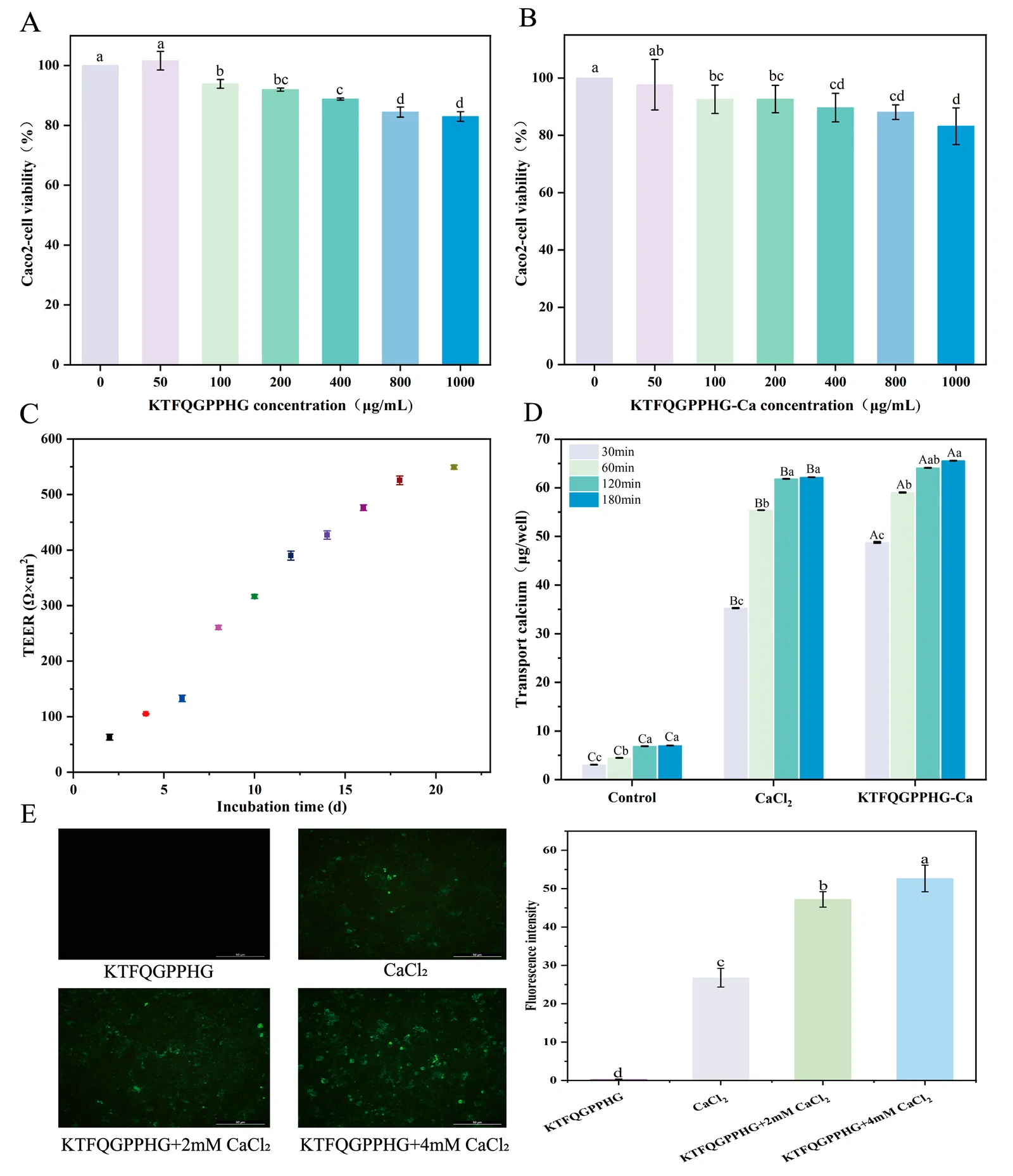
Calcium transport and uptake in Caco-2 cells: (**A**) cytotoxicity of KTFQGPPHG; (**B**) cytotoxicity of KTFQGPPHG-Ca; (**C**) trans-epithelial electrical resistance (TEER) in Caco-2 cell monolayer model; (**D**) calcium transport capacity; (**E**) calcium fluorescence imaging in Caco-2 cells. Different superscript letters (uppercase or lowercase) indicate significant differences between groups (*p* < 0.05).

Figure 5A,B illustrates the cytotoxicity test results. When Caco-2 cells were treated with KTFQGPPHG at 0–1000 μg/mL concentrations, initial proliferation occurred. As the sample concentration increased, cell viability exhibited a decreasing trend. Subsequently, with further elevation of KTFQGPPHG concentration, Caco-2 cell viability consistently remained above 80%. Similarly, cell viability decreased gradually with increasing KTFQGPPHG-Ca dosage (0–1000 μg/mL). Nevertheless, the cell viability still remained above 80%. These results indicate that neither KTFQGPPHG nor KTFQGPPHG-Ca exhibits cytotoxicity toward Caco-2 cells at concentrations from 0 to 1000 μg/mL.

The magnitude of the TEER value reflects the integrity and tightness of the Caco-2 cell barrier [63]. As shown in Figure 5C, the electrical resistance of the Caco-2 cell model increased steadily over 21 days, stabilizing at 526 Ω·cm^2^ on day 18. This indicates that the Caco-2 cells formed a compact monolayer structure suitable for calcium ion transport experiments [64]. Figure 5D shows the impact of KTFQGPPHG-Ca and CaCl_2_ on calcium transport in a monolayer of Caco-2 cells. The amount of calcium transported in both the KTFQGPPHG-Ca group and the CaCl_2_ group showed a significant increasing trend with prolonged incubation time (*p* < 0.05). Between 30 and 120 min, both groups exhibited the maximum calcium transport rate. KTFQGPPHG significantly enhanced calcium transport versus the CaCl_2_ group at 30 min and 60 min (*p* < 0.05), with 1.4-fold and 1.1-fold increases. This indicates that the addition of KTFQGPPHG promotes calcium transport in Caco-2 cells. In addition, the calcium transport in both the CaCl_2_ and KTFQGPPHG-Ca groups reached a peak at 180 min, with values of 62.16 μg/well and 65.56 μg/well, respectively. At this time point, KTFQGPPHG-Ca showed 1.05-fold higher calcium transport than the CaCl_2_ group. Moreover, KTFQGPPHG-Ca showed higher calcium transport than CaCl_2_ over the whole duration. In summary, these results reveal that KTFQGPPHG-Ca exhibits significantly superior calcium transport capacity to CaCl_2_ in the Caco-2 model, indicating that KTFQGPPHG enhances calcium transport at the cellular level.

#### 3.4.2. Calcium Fluorescence Imaging in Caco-2 Cells

The calcium uptake by Caco-2 cells treated with KTFQGPPHG at different Ca^2+^ concentrations was visualized under an inverted fluorescence microscope. Fluorescence images of Caco-2 cells exposed to various treatments are presented in Figure 5E. Both the fluorescence intensity and fluorescence area exhibited a concentration-dependent relationship with Ca^2+^. Relative to the CaCl_2_ group, both the 0.4 mg/mL KTFQGPPHG + 2 mM CaCl_2_ and 0.4 mg/mL KTFQGPPHG + 4 mM CaCl_2_ exhibited stronger fluorescence signals, displayed more fluorescence, with significantly enhanced fluorescence intensity (*p* < 0.05). This indicates that the addition of KTFQGPPHG facilitates the influx of more Ca^2+^ into Caco-2 cells. Furthermore, the intracellular fluorescence in the 0.4 mg/mL KTFQGPPHG + 4 mM CaCl_2_ group was markedly more intense than that in the CaCl_2_ group and the 0.4 mg/mL KTFQGPPHG + 2 mM CaCl_2_ group (*p* < 0.05). This suggests that intracellular fluorescence intensity may increase with increasing CaCl_2_ concentration, as more Ca^2+^ enters the cells. These results imply that, relative to the CaCl_2_ group, the addition of KTFQGPPHG promotes Ca^2+^ influx into Caco-2 cells. Additionally, as the Ca^2+^ concentration increases, the amount of Ca^2+^ entering the Caco-2 cells increases, and the fluorescence intensity also increases significantly (*p* < 0.05). In conclusion, KTFQGPPHG-Ca effectively enhances the calcium uptake capacity of Caco-2 cells.

#### 3.4.3. Calcium Uptake Under the Influence of Dietary Inhibitory Factors

**Figure 6:**
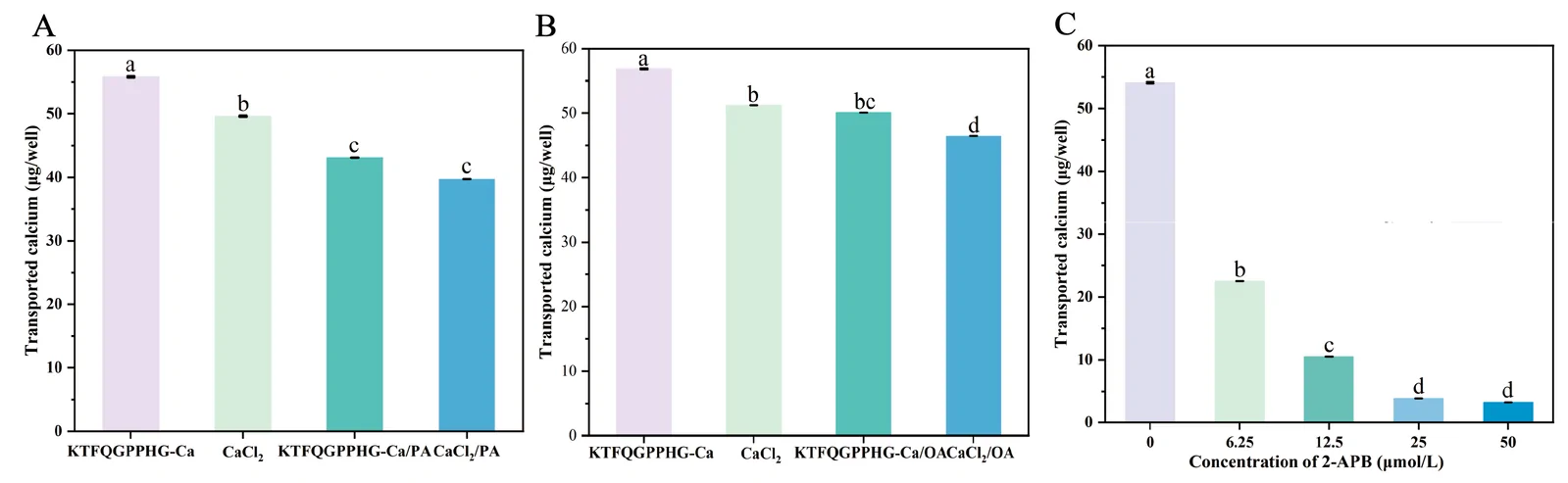
Effect of KTFQGPPHG on calcium transport in Caco-2 cells: (**A**) effect of KTFQGPPHG-Ca on calcium uptake in the presence of phytic acid; (**B**) effect of KTFQGPPHG-Ca on calcium uptake in the presence of oxalic acid; (**C**) effect of the TRPV6 inhibitor 2-APB on calcium uptake. Different superscript letters indicate significant differences among groups (*p* < 0.05).

Food contains various dietary factors such as phytic acid, oxalic acid, and cellulose, which readily form precipitates with Ca^2+^, reducing calcium solubility and thus lowering its bioavailability, which may not meet human calcium supplementation requirements [65]. As shown in Figure 6A,B, in the presence of these dietary inhibitors, the cellular calcium transport in both the KTFQGPPHG-Ca and CaCl_2_ decreased markedly (*p* < 0.05). With the addition of phytic acid, the calcium transport of KTFQGPPHG-Ca was 43.07 μg/well, whereas that of CaCl_2_ was 39.74 μg/well. With the addition of oxalic acid, the calcium transport of KTFQGPPHG-Ca was 50.06 μg/well, compared with 46.48 μg/well for CaCl_2_. In vitro cell experiments demonstrated that both oxalic acid and phytic acid exhibited inhibitory effects on calcium transport in both CaCl_2_ and KTFQGPPHG-Ca groups. Notably, compared with CaCl_2_, KTFQGPPHG-Ca exhibits superior calcium transport capacity, indicating that KTFQGPPHG-Ca may inhibit the precipitation of calcium with phytic acid or oxalic acid, thereby alleviating the adverse effects of unfavorable food components on calcium absorption. This is consistent with the results reported by Wu et al. [66] regarding dietary factor inhibition of octopus peptide–calcium. In summary, these findings suggest that KTFQGPPHG-Ca possesses strong calcium-binding ability and resistance to precipitation by phytic acid and oxalic acid, making it a promising mineral carrier for efficient calcium utilization in the human body.

#### 3.4.4. Effect of TRPV6 Pathway Inhibitor on Calcium Transport

Calcium can achieve transcellular transport through the action of the TRPV6 calcium channel. Transcellular calcium transport is primarily mediated by the TRPV6 calcium ion channel. Ca^2+^ enters the cell through the TRPV6 channel, interacts with Calbindin-D9k for intracellular delivery to the basolateral membrane, and is subsequently released into the bloodstream via PMCA1b [67]. The membrane-permeable compound 2-APB is a small-molecule inhibitor of TRPV6 and has been shown to modulate the function of various ion channels, including two-pore potassium channels and calcium release-activated channels [68]. In Figure 6C, the suppressive effect of 2-APB on cellular calcium influx was examined. Versus the control group, the calcium transport of the KTFQGPPHG-Ca group decreased notably to 22.52 μg/well after the addition of 6.25 μmol/L 2-APB. When the concentration of 2-APB was increased to 25 μmol/L, the calcium transport further decreased significantly to 3.88 μg/well (*p* < 0.05). This indicates that the TRPV6 channel may be involved in the process by which KTFQGPPHG-Ca enhances calcium intake in Caco-2 cells. In conclusion, KTFQGPPHG-Ca may facilitate Ca^2+^ transport through the TRPV6 calcium channel.

## 4. Conclusions

In this study, a novel calcium-binding peptide, KTFQGPPHG, was successfully identified from *Moringa oleifera* Lam. leaf protein hydrolysate and further prepared as a peptide–calcium chelate, KTFQGPPHG-Ca, which exhibited strong calcium-binding capacity. Structural characterization confirmed that calcium ions form stable chelates with KTFQGPPHG through carboxyl oxygen and amino nitrogen atoms. This chelation mode is highly similar to the coordination modes of mung bean-derived calcium-binding peptides reported by Zhai et al. [4] and walnut-derived peptides identified by Wang et al. [69]. Therefore, carboxyl oxygen and amino nitrogen appear to be common coordination sites universally involved in the chelation of calcium ions by peptides from different sources. Notably, the amino acid sequence specificity of the peptide itself also influences the specific calcium-binding mode [70]. In this study, structural characterization only clarified the types of atoms involved in chelation; however, the specific amino acid residues of KTFQGPPHG that participate in calcium ion binding have not been fully elucidated. Future studies could employ molecular dynamics simulations to further quantify the binding free energy between the peptide and calcium ions and the contributions of key residues. After chelation, the peptide transformed from an amorphous structure to a stable crystalline structure, significantly enhancing its structural stability. Studies on bamboo shoot peptide–calcium chelate also found that the addition of calcium ions altered the surface morphology, crystal structure, and secondary structure of the peptide, inducing a transition from a disordered structure to an ordered structure [71]. This demonstrates that the introduction of calcium ions may induce ordered folding of the peptide chain and intermolecular rearrangement, and such changes may enhance the structural stability of the peptide–calcium chelate. Stability analysis demonstrated that KTFQGPPHG-Ca possesses favorable thermal stability, phosphate stability. This is consistent with most reported peptide–calcium chelates. Chelates derived from various peptide sources commonly possess good thermal tolerance and phosphate stability [72]. This may be attributed to the fact that the chelated state formed via coordination interactions can effectively protect calcium ions and prevent their precipitation under heat treatment and phosphate-containing conditions. In vitro cell experiments demonstrated that KTFQGPPHG-Ca exhibits efficient calcium transport in Caco-2 cells, and partially counteract the inhibitory actions of dietary antinutritional factors such as phytic acid and oxalic acid on calcium absorption. Hou et al. [24] also reported that desalted duck egg-white peptides could mitigate calcium precipitation induced by dietary factors, which is consistent with our present findings. The presence of peptides may help maintain higher calcium bioavailability in the gastrointestinal tract [73]. In addition, our results indicated that the calcium transport pathway of KTFQGPPHG-Ca potentially involves the TRPV6 calcium channel. TRPV6 is a key channel for active transcellular calcium absorption in the intestine. It governs the rate-limiting step for calcium influx into intestinal epithelial cells and plays an essential role in maintaining systemic calcium homeostasis under low calcium intake conditions. Nevertheless, KTFQGPPHG-Ca does not rely solely on the TRPV6 calcium pathway for calcium transport. Our other study reveals that the calcium absorption-promoting effect of KTFQGPPHG-Ca may involve additional pathways, and multiple pathways can work synergistically to facilitate calcium uptake [12]. Previous studies have reported that food-derived peptide–calcium systems participate in calcium absorption through the TRPV6 calcium channel pathway [74]. In comparison, our validation of the TRPV6-related mechanism is primarily based on channel inhibitor experiments, without direct molecular-level proof. Further molecular–biological investigations including gene-expression assays are needed to confirm this mechanism. In summary, this study provides a new approach for the high-value utilization of *Moringa oleifera* Lam. leaf protein and also provides a theoretical basis and candidate molecules for the development of novel peptide–calcium supplements.

## Figures and Tables

**Table 1:** The top ten peptides with the highest molecular docking scores for TRPV6.

Sequence	Intensity Value	*M*_w_	Score	Binding Energy (kcal/mol)
KTFQGPPHG	41,932,000	967.4876	133.7	–9.3
GWLGHPIF	665,495,000	925.481	102.3	–9.2
FEFPAVDT	103,563,600	924.4229	83.61	–8.7
VVDLLAPY	1,007,546,000	888.4957	80.38	–8.7
PWGNAPGA	30,095,300	768.3555	62	–8.7
GHPWGNAPG	2,903,680,000	891.3988	85.31	–8.6
RAPWLEPI	1,117,260,000	980.5444	97.6	–8.4
GHPWGNAPGA	7,408,900,000	962.4359	99.38	–8.4
AKVIHDRF	342,704,000	984.5505	107.9	–8.3
DWVSLPGV	128,509,000	871.444	87.63	–7.8

## Data Availability

The original contributions presented in this study are included in the article/Supplementary Materials. Further inquiries can be directed to the corresponding authors.

## Supplementary Materials

The following supporting information can be downloaded at https://www.mdpi.com/article/10.3390/foods15193496/s1, Figure S1: Morphological characteristics and taxonomic position of *Moringa oleifera* Lam. (A) Whole plant of *Moringa oleifera* Lam.; (B) leaves; (C) flowers, pods and seeds; (D) taxonomic hierarchy. Figure S2: Chromatograms of KTFQGPPHG and GWLGHPIF peptide sequences.
