# Supplementary material for: Moringa oleifera Lam. Leaf Peptide–Calcium Chelates: Structural Characterization, Stability and Calcium Absorption-Promoting Transport Capability
Source: Foods. 2026 Sep 29;15(19):3496. doi: 10.3390/foods15193496 (PMC13664339; doi:10.3390/foods15193496)
Supplement: Supplementary file 1 [file foods-15-03496-s001.zip › foods-4492214-supplementary.pdf]

Supplementary material

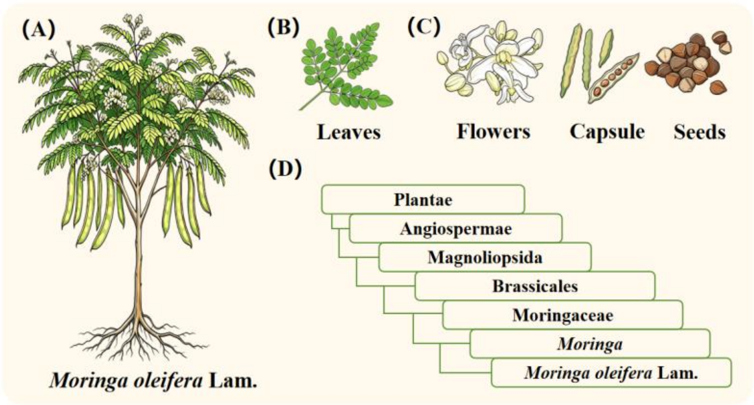

Figure S1. Morphological characteristics and taxonomic position of *Moringa oleifera* Lam. (A) Whole plant of *Moringa oleifera* Lam.; (B) Leaves; (C) Flowers, pods and seeds; (D) Taxonomic hierarchy.

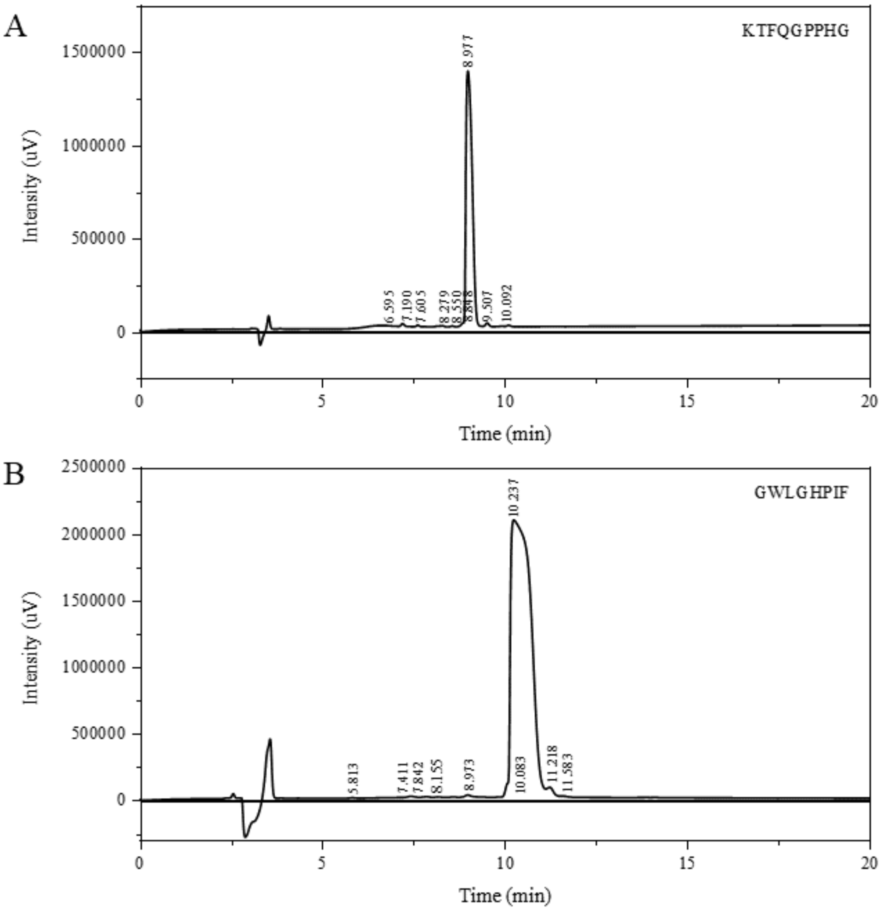

Figure S2. Chromatograms of KTFQGPPHG and GWLGHPIF peptide sequences
